# Ethical Issues in Empirical Studies on Smart Home Technologies: A Scoping Review

**DOI:** 10.1093/geroni/igaf122.1193

**Published:** 2025-12-31

**Authors:** Agnes Jihae Kim, Taekyung Kim, Jiwon Park, Moon Choi

**Affiliations:** Korea Advanced Institute of Science & Technology, Daejeon, Republic of Korea; Korea Advanced Institute of Science & Technology, Daejeon, Republic of Korea; Korea Advanced Institute of Science & Technology, Daejeon, Republic of Korea; Korea Advanced Institute of Science & Technology, Daejeon, Republic of Korea

## Abstract

Smart home technology is increasingly employed to enhance independence, participation, and healthcare for older adults and individuals with disabilities. Given its close integration into daily life, ethical concerns surrounding these technologies have been widely discussed. However, limited research has examined how ethical considerations are applied in empirical studies and how existing conceptual models inform their use. This scoping review provides a comprehensive analysis of the integration of ethical frameworks in empirical studies on smart home technologies for these populations. Following PRISMA-ScR guidelines, two databases (Web of Science and SCOPUS) were searched, identifying 373 articles after duplicate removal. Using Covidence software, reviewers screened articles in pairs, resulting in 37 eligible studies. The findings indicate that 15 studies focused on smart home technologies for social or healthcare applications, with integrated systems being the most prevalent technology type, identified in 20 studies. Privacy was the most frequently addressed ethical issue, appearing in 34 of 37 studies. While privacy concerns vary based on user characteristics and context, broader ethical considerations—including autonomy, trust, bias, and stigma—remain underexplored. Moreover, ethical concerns evolve across different stages of technology development, yet relatively few studies address these issues in early phases. This review highlights the need for a systematic and comprehensive approach to ethical considerations in smart home technology research. It moves beyond theoretical discussions to examine how ethical issues are addressed in the actual processes of developing, adopting, and using smart home technologies based on empirical evidence.

